# Dynamic Regulation of NF-κB Response in Innate Immunity: The Case of the IMD Pathway in Drosophila

**DOI:** 10.3390/biomedicines10092304

**Published:** 2022-09-16

**Authors:** Alexandre Cammarata-Mouchtouris, Adrian Acker, Akira Goto, Di Chen, Nicolas Matt, Vincent Leclerc

**Affiliations:** 1 Institut de Biologie Moléculaire et Cellulaire (IBMC), UPR9022, CNRS, Université de Strasbourg, 67084 Strasbourg, France; 2 Sino-French Hoffmann Institute, School of Basic Medical Science, Guangzhou Medical University, Guangzhou 511436, China

**Keywords:** innate immunity, *Drosophila* model, NF-κB pathways, dynamic regulation, epigenetics

## Abstract

Metazoans have developed strategies to protect themselves from pathogenic attack. These preserved mechanisms constitute the immune system, composed of innate and adaptive responses. Among the two kinds, the innate immune system involves the activation of a fast response. NF-κB signaling pathways are activated during infections and lead to the expression of timely-controlled immune response genes. However, activation of NF-κB pathways can be deleterious when uncontrolled. Their regulation is necessary to prevent the development of inflammatory diseases or cancers. The similarity of the NF-κB pathways mediating immune mechanisms in insects and mammals makes *Drosophila melanogaster* a suitable model for studying the innate immune response and learning general mechanisms that are also relevant for humans. In this review, we summarize what is known about the dynamic regulation of the central NF-κB-pathways and go into detail on the molecular level of the IMD pathway. We report on the role of the nuclear protein Akirin in the regulation of the NF-κB Relish immune response. The use of the *Drosophila* model allows the understanding of the fine-tuned regulation of this central NF-κB pathway.

## 1. *Drosophila melanogaster*: A Case Study of the Innate Immune System

### 1.1. Introduction

The immune system is composed of tissues, cells and molecules within an organism that collectively aim to detect agents that are different from the organism’s healthy tissues and organize a response to counteract them and maintain homeostasis. There are two main types of systems: innate and adaptive. The innate immune system precedes the adaptive response and presents conserved mechanisms [1]. It involves a variety of cells and molecular pathways to mount a fast immune response [2,3,4].

The innate immune pathways mainly involve three types of proteins: sensors, which are able to detect microbial patterns or danger signals; adaptors, which are able to transduce the signal downstream of the signaling pathway; and effectors and regulators, which are crucial to the immune response and its dynamicity. When abnormally regulated, innate immune responses contribute to the development of pathologies including chronic inflammation, autoimmune diseases and cancer [5,6]. The notion of intrinsically dynamic regulation proves to be essential in the understanding of the innate immune system.

### 1.2. Drosophila: An Ever-Relevant Model

*Drosophila* are a well-suited model to unravel the fundamental mechanisms that constitute the innate immune response. They share many molecular pathways underlying the activation of their innate immune systems with humans [7], and studies have demonstrated the relevance of the model in this context [8]. On a similar note, several studies present *Drosophila* as a model insect in the field of oncology [9,10,11,12]. In cancer research, flies have been crucial to the discovery of genes and pathways that play oncogenic roles [13,14,15].

The flies present cellular local responses [16] and a systemic immune response. Activation of the second type of response relies on nuclear factor kappa B (NF-κB) pathways. There are two pathways, named immune deficiency (IMD) and Toll, with distinct specificities and characteristics [17,18]. The architecture of the pathways is conserved in mammals, with a strong similarity between IMD and TNFα pathways and between Toll and TLR pathways.

The simplicity of the *Drosophila* system in respect to mammals allowed the identification or analysis of processes and molecules, as exemplified by the Nobel Prize attributed to Jules A. Hoffmann and Bruce A. Beutler in 2011 for their discovery of the role of Toll receptors in the activation of the innate immune response [19]. It is therefore of great importance to keep an eye on flies’ NF-κB: it is still moving!

## 2. Overview of the NF-κB Signaling Pathways in *Drosophila*

NF-κB was originally identified as a DNA-binding activity protein in activated B cells [20]. There are two NF-κB pathways in *Drosophila* that play a fundamental role in their immune response. The IMD and Toll pathways are able to recognize three main pathogen families: mostly Gram-negative bacteria for IMD, Gram-positive bacteria and fungi for Toll [21].

### 2.1. The IMD Pathway

**Figure 1:**
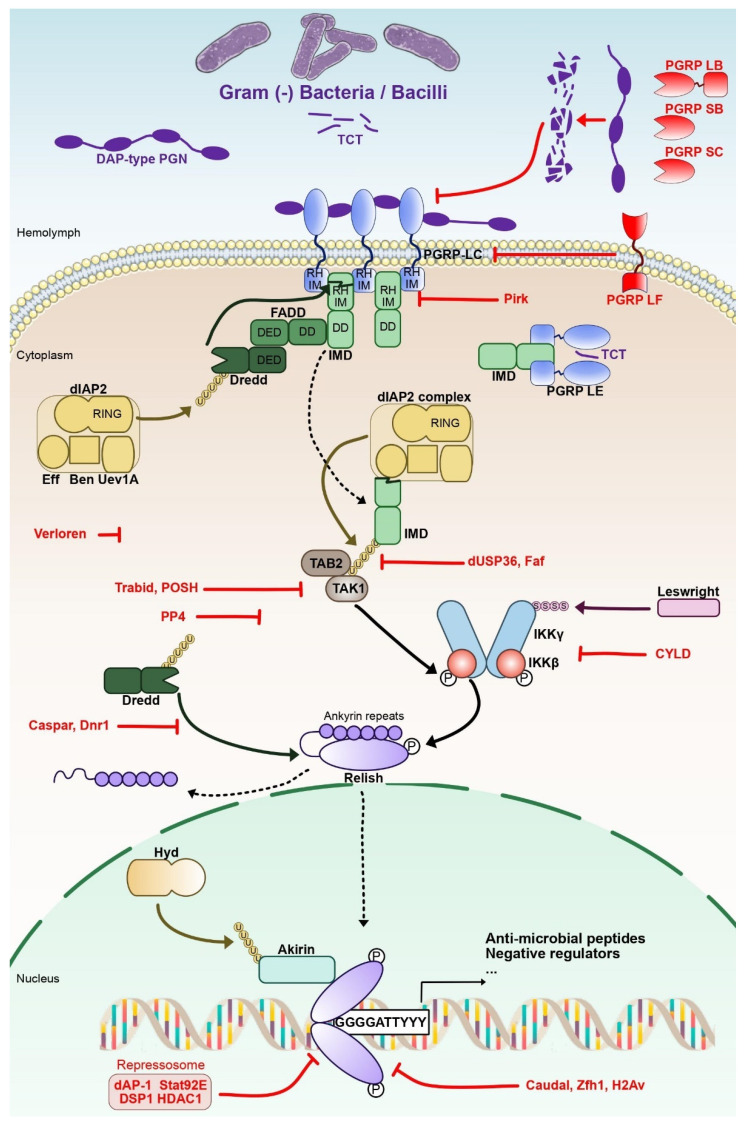
**The NF-κB IMD pathway in *Drosophila*.** IMD is activated through the recognition of Gram-negative bacteria-derived meso diaminopymelic-type (DAP-type) peptidoglycan (PGN) and tracheal cytotoxin (TCT) by the Peptidoglycan recognition (PGRP) domain of Peptidoglycan recognition protein -LC and -LE (PGRP LC, -LE). PGRP-LC and -LE death-domains recruit immune deficiency (IMD), FAS-associated death domain (FADD) and death-related ced-3/Nedd2-like protein (Dredd). A ubiquitin-ligase complex formed by the E3 ubiquitin ligase Drosophila inhibitor of apoptosis 2 (DIAP2) and the E2 ubiquitin conjugating Ubiquitin conjugating enzyme variant 1A (Uev1a), Bendless and Effete activates Dredd by K63-linked poly ubiquitinylation. Activated Dredd cleaves IMD N-terminal domain, leading to the recruitment of transforming growth factor beta (TGF-β)-activated kinase 1 (TAK1) and TAK1-associated binding protein 2 (TAB2). TAK1 is able to activate the inhibitor of NF-κB (IκB) kinase (IKK) complex formed of IKKβ and IKKγ subunits. Phosphorylated IKKβ is sumoylated by Leswright and phosphorylates the N-terminal portion of the NF-κB factor Relish to enable its transcriptional activity. Relish is separated from its IκB-like C-terminal ankyrin repeat region by Dredd through proteolytic cleavage. The NLS-containing N-terminal portion of Relish (Rel-68) is then imported to the nucleus while the IκB-like C-terminal portion (Rel-49) remains in the cytoplasm. Rel68 homodimers bind their cognate κB Response element, the consensus sequence 5′-GGGGATTYYY-3′ (Y: C or T) and activate IMD-pathway target genes with the help of the nuclear protein Akirin, which needs to be ubiquitinated by the E3-ligase Hyd beforehand. Dotted arrows indicate the activity of key cleaved proteins of the pathway (IMD, Relish), while continuous arrows are used for the other proteins. Negative regulators are highlighted in red.

Microbial-associated molecular patterns (MAMP) of Gram-negative and some Gram-positive bacteria activate the IMD pathway (Figure 1). Meso-diaminopymelic-type (DAP-type) peptidoglycan is linked to two pattern recognition receptors (PRR) of the peptidoglycan-recognition protein (PGRP) family, PGRP-LC and PGRP-LE [22]. In *Drosophila*, at least 13 genes encode 17 PGRPs isoforms through alternative splicing [23].

The activated receptors PGRP-LC or -LE will interact with the protein adaptor IMD. The cleaved N-terminal of IMD exposes an Inhibitor of Apoptosis 2 (IAP2) binding motif (IBM). That will lead to the recruitment of a tetrameric protein complex composed of DIAP2, Ubiquitin-conjugating variant 1a enzyme (Uev1a), Bendless and Effete complex [24]. The role of this complex is to add Lysine 63 (K63)-linked ubiquitin chains on cleaved IMD, leading to recruitment of the Mitogen-associated protein (MAP) kinase kinase kinase (MAPKKK), Transforming growth factor β (TGF-β)-activated kinase 1 (TAK1) and TAK1-associated binding protein 2 (TAB2) [25]. This complex activates the Inhibitor of NF-κB Kinase (IKK). Composed notably of the catalytic subunit IKKβ (or immune-response deficient 5–Ird5) and the regulatory subunit IKKγ (or Kenny–Key), the IKK complex mediates the phosphorylation and proteolytic cleavage of the NF-κB factor Relish. Relish presents a C-terminal Inhibitor of NF-κB (IκB)-like domain that will remain in the cytoplasm following the cleavage and a N-terminal domain that will translocate from the cytoplasm to the nucleus [26,27,28,29,30].

In the nucleus, Relish proteins form homodimers that control the expression of hundreds of target genes, affecting various immune functions such as microbial recognition, melanization or production of reactive oxygen species [31,32,33]. Some of these genes code for anti-microbial peptides (AMPs), small secreted peptides that play a central role in the defense against micro-organisms [34]. Among the Relish NF-κB target genes, negative regulators are expressed to fine-tune the activation and shutdown of the IMD pathway (Figure 1). Those regulatory proteins can be found at different levels of the pathway: during the DAP-type PGN recognition, at the IMD-IKK signaling platform, for Relish cleavage and activity in the nucleus [35,36,37,38]. For instance, Pickle is a nuclear IκB that interacts with the NF-κB protein Relish, selectively repressing Relish homodimers. Loss of Pickle results in over-expression of Relish target genes. Host resistance to pathogenic bacteria improved in the short term, but chronic inactivation of Pickle shortened the lifespan [39].

### 2.2. The Toll Pathway

The Toll pathway is activated upon the sensing of fungi, Gram-positive bacteria and some Gram-negative bacteria. The transmembrane receptor Toll is activated by an extracellular proteolytic signaling cascade in two ways: circulating PRR recognizes Lys-type peptidoglycan (Lys-PGN) from Gram-positive bacteria or β-glucans from fungi [40]; proteases produced by fungi, Gram-positive bacteria and some Gram-negative bacteria are sensed by the proteolytically activable serine protease Persephone (Psh), initiating the “danger-signal” pathway [41,42,43].

The extracellular signaling cascade leads to activation of Spätzle (Spz) and its binding to Toll. This initiates the internalization of the receptor and recruitment of the adaptor protein Myeloid differentiation primary response gene 88 (MyD88) through their common TIR domains [44,45,46,47]. MyD88 recruits a secondary adaptor, Tube, through its death domain (DD), leading to the formation of a tripartite complex with Pelle, a kinase homolog to the mammalian Interleukin-1 receptor associated kinase 1 (IRAK1). Pelle phosphorylates the Ankyrin-repeats containing IκB-like protein Cactus [46,47,48,49,50]. The subsequent degradation of Cactus by the proteasome releases the NF-κB factors Dorsal or Dorsal-related immunity factor (DIF). They translocate to the nucleus and exert their DNA-binding activity [51,52,53,54]. Nine Toll-related receptors (Toll-1 to -9) have been identified, with Toll (also called Toll-1) being the main receptor for NF-κB-dependent AMPs synthesis [55]. The principal Toll AMPs are the anti-Gram-positive bacterial Defensin and the antifungal Drosomycins and Metchnikowin [56,57]. Upon activation of both IMD and Toll pathways, formation of heterodimers of Relish and DIF or Dorsal leads to both IMD and Toll pathway target gene expression [58].

Toll pathway activation must also be controlled to prevent putative harmful activations. Only one negative regulator has been identified up until now: Pellino, which regulates Myd88 protein stability [59]. While this work is in contradiction with a previous study that showed Pellino’s requirement for Toll signaling [60], this protein is part of the only feedback regulatory loop described in the Toll pathway that prevents excessive activation. We hope that further work will help characterize better the regulation of the NF-κB factor DIF activity and the differences with the regulation of Relish.

### 2.3. Regarding Both Pathways

A difference between the two pathways is the timing of activation: the IMD pathway is rapidly activated within minutes in cell cultures and AMP gene expression culminates around 6 h post stimulation, whereas the Toll pathway takes longer to get activated, with a peak of gene expression around 24 h post stimulation. We have no clue if this is due to differences in the mode of activation of the pathways or something else.

Recent work in various laboratories provides a more comprehensive view of the IMD pathway, its regulation and the dynamic control of gene expression by its NF-κB factor, Relish. We decided to focus the rest of the review on what is known about Relish regulation as a case study to understand the regulation of NF-κB response.

## 3. From PAMP Sensing to NF-κB Relish Activity

### 3.1. PGRP-LC/PGRP-LE-IMD Signaling Complex

Activation of the IMD pathway requires the formation of a high molecular weight protein complex, described as a signaling amyloid complex. Through homophilic interactions between proteins bearing the same homology domain and poly-ubiquitin chains, the various proteins of the pathway aggregate, allowing the activation of Relish. This process is detailed in [61] and can be summarized as follows.

Binding of DAP-type peptidoglycan to PGRP-LC at the cell membrane or to PGRP-LE in the cytosol leads to multimerization of the receptor and activation of the pathway (Figure 1). Given that overexpression of the receptors is sufficient to activate the pathway, this prompted the suggestion of the proximity hypothesis. PGRP-LC and PGRP-LE intra-cytosolic domains contain a RIP homotypic interaction domain (RHIM) that misses a conserved Gln residue and is therefore called a cryptic-RHIM (cRHIM). This cRHIM is nevertheless able to form amyloid by aggregation of the domains and formation of a hydrophobic core. The aggregation of several receptor molecules is supposed to be sufficient for the recruitment of IMD, which also contains a cRHIM. This induces the formation of the IMD amyloid [62]. Relish also contains a cRHIM but it is not known whether it is recruited to IMD amyloid.

### 3.2. From IMD to Relish Activation

The C-terminal domain of IMD contains a death domain (DD) that interacts with the DD of FADD (see [32] for a review). FADD recruits the caspase-8 DREDD, which is not activated by cleavage: DREDD recruits the ubiquitin ligase Diap2, which leads to the K63-polyubiquitination of DREDD and its activation. DREDD cleaves Relish and releases the ankyrin repeat domain. DREDD also cleaves the N-terminal domain of IMD, allowing Diap2 mediated K63-polyubiquitination of IMD and the recruitment of Tab2 and the kinase TAK1 [63]. TAK1 phosphorylates the IKK complex (IKKγ Kenny and IKKβ Ird5 kinase). Kenny may be recruited through binding to K63-polyubiquitin but it has not been shown yet. Ird5 phosphorylates Relish.

The activation of DREDD is therefore responsible for the two modifications of Relish required for its activation. Cleaved and phosphorylated Relish is the active form of the transcription factor able to enter the nucleus. It has not been shown yet where the phosphorylation takes place and what its exact function is. If Relish is recruited to the amyloid through its cRHIM, it could be involved in releasing Relish from the amyloid.

### 3.3. Molecular Mechanism of Relish Activation

In the absence of any stimulus, NF-κB transcription factors are sequestered in the cytoplasm by interacting with an ankyrin-repeat-containing protein. All NF-κB proteins share a N-terminal domain, the Rel homology domain (RHD), which is responsible for binding to DNA and homo or heterodimerization [64]. There are two classes of NF-κB factors: the first ones are *Drosophila* Dorsal or DIF, which are homologous to mammalian RelB or p65/RelA. They contain a C-terminal transactivation domain and associate with an ankyrin-repeat containing protein of the IκB family, such as Cactus. Another class is Relish, which is the homolog of mammalian p100 or p105.

In the case of Relish, instead of association with the other ΙκB family genes, such as Cactus, Relish itself contains a C-terminal ankyrin-repeat domain. Thus, when Relish gets activated, it must be phosphorylated and cleaved in order for the transcription factor domain to be able to enter the nucleus. The complexity of the IMD pathway is related to this complex mode of activation of Relish.

### 3.4. Complex of Transcription Factors

After its cleavage, the Relish N-terminal domain (RelN) is translocated to the nucleus, which opens the possibility of target gene transcriptional activation, but only if Relish is allowed to access its targets (Figure 1).

Ecdysone is a developmental hormone required for metamorphosis. It is sensed by a nuclear receptor, which activates some early response genes such as the Broad-complex (BR-C). In both S2 cells [65] and Malpighian tubules [66], BR-C has two functions: it stimulates the expression of genes coding for proteins of the IMD pathway, such as Relish or PGRP-LC; and it interacts with Relish and increases the response of some of Relish’s target genes [65]. Both functions result in an increased response of the cells to infection and resistance of flies. We can suppose that Ecdysone induces the maturation of adult cells, which includes the responsiveness of immune cells, and that Relish activity depends on the presence of some transcription factors on the regulatory sequences of target genes. Indeed, BR-C binding sites are found on several AMP-coding genes.

Furthermore, several other transcription factors, such as the GATA factors Pannier and Serpent, are required in addition to BR-C for the immune response in S2 cells, in the fat body and for the survival of flies after infection [65]. One hypothesis could be that those factors participate in the identity of the cells and are part of a combination of transcription factors, including Relish, required for expression of target genes. Whether they are required for Relish to access the DNA remains to be determined.

### 3.5. Other Ways to Activate NF-κB Relish Pathway

Other than downstream of the IMD pathway, Relish can also be activated in different circumstances by mechanisms that are not well understood. It is expressed as a response to hypoxia and the activation of FOXO transcription factor [67], but we do not know how it is activated in this case, nor if the whole repertoire of target genes is the same. Relish is also activated as an antiviral response after sensing of 2′3′cGAMP by dSting [68,69]. Relish activation, in this case, is dependent on IKKβ but not IKKγ and activates a different set of genes than after activation of the IMD pathway. This could be due to the presence of another transcription factor.

Our laboratory in Strasbourg recently conducted a study focused on the response of drosophila adult flies to oncogenic RasV12-expressing cells injected into the body cavity [70]. Eleven days after injection, we noticed that numerous immune-induced genes were activated. Whether this is due to the activation of an NF-κB transcription factor and how it could be linked to the presence of cancerous cells remains under investigation.

## 4. Various Ways to Inactivate the NF-κB IMD-Relish Pathway

Different mechanisms are involved in keeping the pathway silent in the absence of infection, in tolerance to commensals or in rapid resolution of the response after an infection through auto-regulatory loops (Figure 1). In those cases, the molecules are involved in both tolerance and resolution when they have been analyzed.

### 4.1. At the Level of the Receptor

The PGRP family of proteins is subdivided into two categories: some, such as PGRP-LC or PGRP-LE, bind the ligand, whereas others, such as PGRP-SC and PGRP-LB, are amidase enzymes that cleave the ligand and are unable to activate the pathway [70]. This second class of molecules is involved in eliminating a moderate amount of PGN, which is important for immune tolerance to commensal bacteria in the gut. In the case of infection, PGN concentration exceeds the capacity of this amidase, allowing activation of the IMD pathway. PGRP-SC family and -LBPC isoforms are secreted molecules that avoid activation of PGRP-LC, whereas PGRP-LBPA/PD isoforms are cytosolic molecules that avoid activation of PGRP-LE [71]. Their deletion reduces the life span due to a low level of IMD pathway activation. Furthermore, they are required to reduce activation of the pathway after an infection [72,73].

PGRP-LF is a transmembrane protein that lacks the intracytosolic RHIM domain. In its absence, the IMD pathway is constitutively activated [37]. PGRP-LF does not bind PGN, but it binds the ectodomain of PGRP-LC [74]. The proposed model is that PGRP-LF inhibits auto-activation of the pathway by preventing functional multimerization of PGRP-LC isoforms. The PGRP-LC receptor gene itself encodes several isoforms through alternative splicing. Regulative PGRP-LC isoforms (rPGRP-LC) intracytosolic domain contains a PHD domain instead of the RHIM domain [75]. It is therefore unable to signal. Furthermore, it is required to resolve IMD pathway activation after an infection. rPGRP-LC binds to polymeric PGNs that are characteristic of dead bacteria and therefore of effective killing. rPGRP-LC induces endocytosis of PGRP-LC and termination of signaling. rPGRP-LC also binds to Pirk and Diap2. Pirk is a negative regulator of the IMD pathway, required in the same way as PGRP amidases to resolve IMD pathway activation after an infection and tolerate commensal [36,38,76,77]. Pirk is rapidly induced after an infection. Pirk contains a cRHIM domain and interacts with the cRHIM domain of PGRP-LC and PGRP-LE. It could act through either destabilization of IMD amyloid [62] or targeting PGRP-LC to degradation [75].

### 4.2. At the Level of the Signaling Cascade

Many key steps of IMD pathway activation involve the deposition of K63 poly-ubiquitin chains. K48 poly-ubiquitinations are known to activate the degradation of proteins. Therefore, it does not come as a surprise that several negative regulators of the pathway have been identified to regulate those processes. As explained, once recruited to the IMD amyloid, TAK1 phosphorylates the IKK complex, leading to Relish activation and IMD itself leading to K63-deubiquitination. Drosophila ubiquitin-specific protease 36 (dUSP36) prevents the accumulation of K63-activated IMD, thus promoting its degradation [78]. Fat facets (faf) is another deubiquitinase that has been shown to negatively regulate the IMD pathway at the level of the IMD protein [79]. TAK1 also induces K48-ubiquitination and proteasome-dependent degradation of IMD [63].

The deubiquitinating enzyme cylindromatosis (CYLD) has been reported to inhibit IMD pathway activation, possibly by targeting the IKK complex [80], which is similar to what is known for the mammalian ortholog of CYLD [81,82]. The IKK complex is also targeted for degradation, but through autophagy and proteasome-dependent proteolysis [83]. The absence of autophagy leads to constitutive activation of the pathway by commensal bacteria, but it was not shown if it is involved in the resolution of the activation after an infection. TAK1 activation also activates the JNK pathway that contributes to the immune response in parallel to the Relish-activated response. Plenty of SH3s (POSH), which is an E3 ubiquitin ligase, negatively regulates the IMD pathway by ubiquitinating TAK1, inducing its degradation [84]. Moreover, the de-ubiquitinase Trabid negatively regulates the IMD pathway at the level of TAK1 as well. Indeed, Trabid decreases the levels of K63 polyubiquitination on TAK1, and Trabid mutant flies exhibit elevated AMP levels and reduced life span due to intestinal dysbiosis [85]. Pvf2 and Pvf3 are induced by the JNK pathway and bind to the Pvr receptor. They dampen TAK1 activity and then contribute to the resolution of the infection [86].

Caspar, a ubiquitin-related domain bearing protein, inhibits the cleavage of Relish by the caspase Dredd [87]. Although the molecular mechanism of Caspar remains unknown, its human ortholog hFAF1 is known to activate the ubiquitin-proteasome pathway [88], suggesting that Caspar could target Dredd for degradation by the proteasome. In addition, Defense repressor 1 (Dnr1) has been proposed to inhibit the activity of Dredd as well, as Dnr1 deficiency results in an over-activation of the IMD pathway both in vitro and in vivo [89,90]. As the RING-finger containing protein Dnr1 physically binds to Dredd, it might inhibit Dredd by directing it to proteasomal degradation, although this has not been formally demonstrated [90].

Similarly to ubiquitination, several phosphorylation reactions also play a pivotal role in IMD pathway signaling. Recently, our group published the identification of the protein phosphatase PP4 as a new negative regulator of the IMD pathway [91]. Inhibition of the components of the PP4 complex induced an over-activation of the IMD pathway, highlighted by a marked increase in AMP levels both in vitro and in vivo, which ultimately leads to a reduced lifespan of adult flies. Conversely, flies over-expressing the negative regulator PP4 exhibit an impaired immune response and are not able to control Gram (-) bacterial infection [91]. The involvement of additional protein phosphatases in the regulation of the IMD pathway is quite likely and remains to be explored.

### 4.3. At the Level of Relish

The transcription factor of the homeobox family Caudal has been shown to negatively regulate the IMD pathway in the intestine (Figure 1). Indeed, caudal deletion induced an increase in AMP levels in the gut, leading to an altered microbiota composition and ultimately to a shortened life span [92]. This negative regulation of Relish target genes could occur through the binding of Caudal to Caudal-protein DNA recognition elements (CDRE) found in the promoter of AMP genes, although it remains to be experimentally confirmed [93]. Zinc finger homeodomain 1 (Zfh1) is another transcription factor identified as a negative regulator of IMD pathway activation [94]. Inhibition of Zfh1 expression elevates IMD pathway activation in a cellular model, while in vivo inhibition of Zfh1 caused an increase in AMP gene expression only for Cecropin B and Attacin A [94]. The precise mechanism by which Zfh1 inhibits the IMD pathway still remains to be clarified.

Additionally, the transcription factors downstream of the JNK and of the JAK/STAT pathways have been shown to negatively influence the transcriptional activity of Relish. Drosophila activator protein 1 (dAP-1) and Stat92E, the specific transcription factors of the JNK and JAK/STAT pathways respectively, have been proposed to form a repressosome complex with a *Drosophila* high mobility group (HMG) protein named Dorsal switch protein 1 (DSP1) [95]. This repressosome complex associates in response to continuous immune signaling to reduce IMD pathway activation. Depletion of either dAP-1, Stat92E or DSP1 was shown to increase AMP expression in vivo, which in turn decreased the survival of the flies after infection, highlighting the deleterious effects of prolonged immune responses. Functionally, the repressosome complex replaced Relish on the AMP gene promoter by recognizing cis-regulatory elements and recruiting histone deacetylase enzymes to inhibit the expression of these genes [95]. The expression of target genes is also blocked by the binding of Nubin, a POU/Oct family transcription factor, to their promoter. In its absence, the Relish targets genes are strongly expressed in both the gut and fat body [96].

The IκB family member Pickle binds to Relish and inhibits its activity, possibly via the recruitment of the histone deacetylase HDAC1. Loss of Pickle results in hyperactivation of the pathway after infection and constitutive activation by commensal bacteria. Pickle is induced in the gut by bacteria in an IMD-independent manner and is not induced after systemic infection [39]. This function of Pickle remains to be verified as some contradictory results have been published for the same protein, also named Charon [97]. Relish activity is also controlled by transglutaminase activity that induces its polymerization and prevents nuclear translocation. The inhibition of transglutaminase results in constitutive activation of the pathway by commensal and increased activation after infection [98]. Transglutaminase is also responsible for the incorporation of polyamine in Relish and inhibition of DNA binding [99], which could also be involved in tolerance to commensal bacteria. There are also indications that Relish is targeted for degradation via binding of dRYPB and ubiquitination [100], which contributes to the resolution of inflammation.

Finally, two studies pointed out the role of SUMOylation in IMD pathway regulation. A small ubiquitin-like modifier (SUMO) is a short ubiquitin-like protein which also covalently attaches to proteins [101]. First, the unconventional histone variant H2Av has been shown to be yet another negative regulator of the IMD pathway, acting at the level of Relish [102]. H2Av mutant larvae show defects in SUMOylation, and the authors identified lysine K823 on Relish as a site for SUMOylation. Moreover, the loss of this SUMOylation site resulted in a constitutive activation of Relish, as Relish SUMOylation prevents its cleavage and activation [102]. In addition, loss of the SUMO-specific protease Verloren led to an over-activation of the IMD pathway, demonstrated by elevated AMP levels both in vitro and in vivo [103]. This defect in regulation of the IMD pathway ultimately reduced the lifespan of the flies and rendered them susceptible to infection by the Gram (-) bacteria *Pseudomonas aeruginosa* [103].

### 4.4. IMD Pathway Modulation: A Highly Dynamic Process

In the quite impressive list of regulators identified so far (Table 1), we observe two kinds of patterns. On one hand, constitutive expression of regulators blocks auto-activation of the pathway but also helps it return to its basal inactive state. Regulators are overwhelmed by infection (i.e., PGRP-SC) or the activation of the pathway (i.e., deubiquitinases or phosphatases). It is surprising that, despite the large number of negative regulators, the inactivation of only one of them generates an auto-activation of the pathway or an absence of resolution. This suggests that the concentration of these proteins is quite low and that any increase in the activation due to the shutdown of a single regulator is sufficient to overcome their overall activity and further activate the pathway. On the other hand, activation-dependent induced expression of regulators can overcome the pathway activation and shut it down. IMD pathway activation is highly dynamic, with a peak of gene expression around 6 h after infection that goes back to undetectable levels of mRNA as soon as 24 h after infection due to the activity of regulators.

Of note, the same regulator can have a constitutive basal expression as well as an induced expression. Pirk and PRGP-LB are good examples of genes whose expressions are rapidly induced by infection in a PGRP-LC-Imd-Relish dependent manner and are involved in the resolution of infection. Pirk and PGRP-LB are also involved in blocking even low levels of activation. Their absence does not lead to a strong systemic activation of the pathway, but to a low level of detectable antimicrobial peptide expression due to commensal bacteria, ultimately affecting the lifespan. It points to the possibility of their expression at a background level when there are not enough elicitors to strongly activate the pathway and express immune effectors, but a sufficient amount to activate some genes, including inhibitors. It prevents a dangerous background level of immune activity and allows tolerance to gut commensal flora. Pickle has a similar effect but its expression is independent of IMD.

Furthermore, commensal bacteria only induce the expression of a subset of genes, including PGRP-SC and PGRP-LB, but not of genes coding for antimicrobial peptides [94]. This suggests that some genes are expressed when the elicitors are in a low concentration, whereas the whole battery of Relish-target genes is expressed in the case of a high concentration of elicitors, corresponding to an infection.

The concomitant expression of effectors and regulators after infection and their differential basal or commensal-activated expression raises the question of the dynamical and precise control of the expression of different classes of genes responding to the same activation pathway.

## 5. Regulation of NF-κB Relish Target Genes Expression

**Figure 2:**
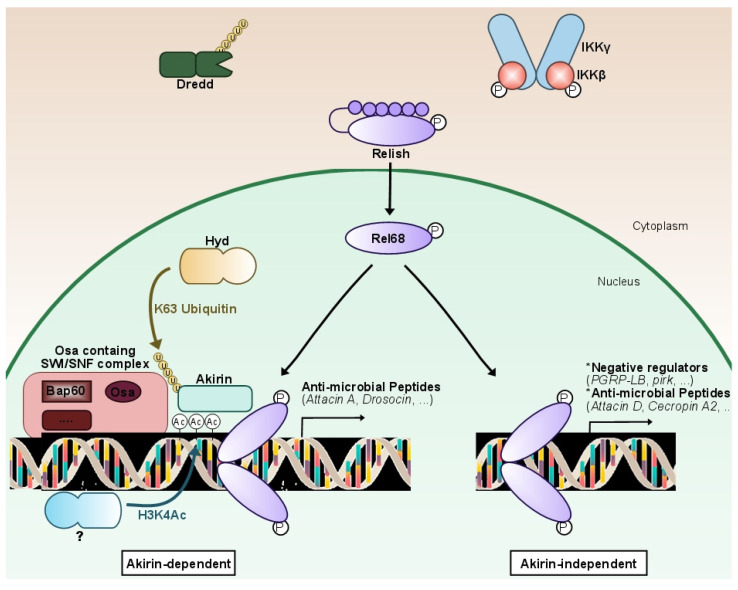
**Fine-tuning characterizes NF-κB IMD pathway expression.** Following the stimulation of the IMD pathway, a non-identified epigenetic-related protein will deposit an acetyl group on the lysine 4 of histone 3 (H3K4Ac), nearby genes mostly coding for effectors (anti-microbial peptides). After being K63 ubiquitinilated by the E3-ligase Hyd, the conserved nuclear protein Akirin orchestrates a NF-κB transcriptional selectivity through the recruitment of the Osa-containing-SWI/SNF-like Brahma complex (BAP). The N-terminal portion of Relish (Rel-68) will then be recruited to the Akirin complex formed, which will lead to the expression of mostly effector genes of the pathway (anti-microbial peptides). In the case that the H3K4Ac mark is not deposited, Akirin will not be recruited. Rel68 will still bind to the consensus sequence and activate the expression of a second subset of genes, comprised of mostly negative regulators and some anti-microbial peptides. In brief, Akirin is a NF-κB co-factor acting as a molecular selector, required for the activation of a specific subset of Relish-dependent genes that correlates with the presence of H3K4Ac epigenetic marks. Akirin specifies the choice between subsets of NF-κB target genes, allowing *Drosophila* to modulate its innate immune response.

The study of the molecular cascade of the IMD pathway in *Drosophila* led to the identification of the nuclear protein Akirin by our laboratory. This evolutionarily conserved player in the NF-κB pathway is required for IMD target gene expression by the Relish transcription factor (Figure 2). Its knock-down in flies leads to a high susceptibility to infections due to the lack of expression of most AMPs [104].

### 5.1. First Discovery of Akirin in Innate Immunity

Akirin was identified by a genome-wide RNAi screening as a positive regulator of the IMD pathway [104]. Knock-down of Akirin in *Drosophila* Schneider 2 (S2) cells reduced the induction of specific IMD pathway regulated antimicrobial peptides, like AttacinA, by 90%. Subsequent epistatic analysis using S2 cells indicated that Akirin acts downstream of or at the level of Relish. Akirin encodes a putative 201 amino acid protein with no recognizable domains but with a clear nuclear localization signal (NLS). This small nuclear protein is highly conserved in metazoan species and consists of one copy in insects and worms and two in vertebrates (except for birds), but none in plants, yeast or bacteria. Complete knockouts of *Drosophila* Akirin and mouse Akirin 2 led to early embryonic lethality at an early or middle stage, but it was not the case in Akirin1 KO mice [105].

Both *Drosophila* Akirin and mammalian Akirin2 are required for the innate immune response. Since Akirins have no obvious DNA binding domain, it was proposed that Akirin could be a cofactor that regulates or fine-tunes NF-κB transcriptional activity by interacting with chromatin remodeling factors and/or the transcriptional machine [104,105,106].

### 5.2. Akirin Fine-Tune the NF-κB Response in Drosophila and Mammals

After stimulation, Akirin is K63-polyubiquitinalyted through the activity of the Hyd E3 ubiquitin ligase [107]. This leads to its binding to Relish, but we do not know how this ubiquitination is triggered. An interesting feature is that Akirin is only required for the activation of a subset of Relish target genes [108]. Indeed, most of Relish-regulated AMP genes are dependent on Akirin, whereas most regulators (including Pirk, PGRP-LB and PGRP-LF) are only dependent on Relish. Some AMP genes are, however, independent of Akirin. We have shown that Akirin-independent gene expression is detected as soon as one hour post-stimulation in cell culture, whereas Akirin-dependent gene expression is only detected at two to three hours.

One interesting hypothesis would be that a moderate or short-time induction of the pathway would only activate, in an Akirin-independent process, a few AMPs to fight infection and most of the regulators of the pathway to maintain the homeostatic state, whereas a strong or prolonged infection would lead to the full Akirin-dependent activation of the pathway, efficient immune response and its subsequent resolution. This would protect against unnecessary activation by commensal or weak infections easily handled by epithelial and phagocytic immune responses. In this model, Akirin-independent genes would be easier and therefore quicker to activate than Akirin-dependent genes. This points to a different epigenetic state of these two classes of genes and a specific function of Akirin to allow activation of less prone-to-activation genes.

As in *Drosophila*, mammalian Akirin-2 acts downstream of the TLR, TNFR and IL-1R signaling pathways [105]. A conditional knockout of *akirin-2* in macrophages compromised the immune response of mice against Listeria monocytogenes intra-peritoneal infections in vivo. Interestingly, mAkirin-2 is required for the regulation of only a subset of LPS and IL-1 inducible genes with mainly pro-inflammatory activity. Moreover, like in *Drosophila*, mAkirin-2 bridges the NF-κB factor and the chromatin remodeling SWI/SNF complex. It also appeared to participate in the innate immune response through its interaction with the nuclear IκB protein IκBζ, an atypical member of the IκB protein family [105].

It was suggested that IκBζ may influence the regulation of histone modification through selective H3K4 tri-methylation of TLR-induced promoters [109]. An increasing number of studies report that IκBζ regulates the activity of the canonical NF-κB p50 transcription [110,111,112]. As there is no homolog of IκBζ in *Drosophila*, another cofactor could be implicated and remains to be identified. Other studies show a NF-κB-dependent immune function of Akirins against Gram-negative bacterial infections or more generally, an indispensable role for the expression of innate immune defense genes [113,114]. These results argue for a conserved role of Akirins to partly regulate the innate immune response of metazoans.

### 5.3. Mechanism of NF-κB Selective Response

Devoid of known predicted functional domains in their sequence, Akirins might gather chromatin-remodeling complexes with sequence-specific targeting transcription factors [115] (Figure 2). Using a genome-wide approach, our laboratory showed that the conserved nuclear protein Akirin is a NF-κB co-factor required for the activation of a subset of Relish-dependent genes, characterized by the presence of the H3K4ac epigenetic mark [108]. This mark is not present on the promoters of the other genes activated by Relish. A large-scale unbiased proteomic analysis revealed that Akirin orchestrates NF-κB transcriptional selectivity through the recruitment of the Brahma-associated protein (BAP) SWI/SNF chromatin-remodeling complex. These findings, conserved from *Drosophila* [108] to mammals [105], link chromatin remodeling to epigenetic control of NF-κB target gene selectivity. Removing Akirin or SWI/SNF leads to an impaired expression of several AMP-coding genes, affecting the innate immune response of *Drosophila* against Gram-negative bacteria and worsening survival after infection [108].

Another group also found a diminution of IMD pathway activation after inhibition of BAP complex genes in cells but, surprisingly, an increase in several IMD genes in vivo [116]. We cannot explain this discrepancy at the moment. Moreover, Akirin has also been shown to bridge the SWI/SNF chromatin remodeling complex to target genes during muscle development, where Akirin is also necessary for the function of the transcription factor Twist [117]. Several epigenetic-related proteins have been identified to physically interact with core members of the IMD pathway [118]. Among them, DMAP1, a member of the Tip60-p400 histone acetyltransferase complex, is necessary for optimal expression of IMD target genes and physically interacts with Relish and with components of the BAP SWI/SNF remodeling complex [119]. Its relation to Akirin is under investigation.

Another insight into the pivotal function of chromatin regulation during inflammation lies in the role of a distinct SWI/SNF chromatin remodeling complex, the Poly-Bromo associated Brahma (PBAP) complex, as a negative regulator of IMD signaling in the gut [120]. Bap 180, a component specific of the PBAP complex, was shown to directly interact with the IMD transcription factor Relish and to be recruited to the promoter regions of antimicrobial peptides regulated by the IMD pathway. Flies mutant for Bap180 show increased susceptibility to infections by Gram-negative bacteria as a result of elevated expression of pro-inflammatory IMD-target genes/anti-microbial peptides in the gut rather than elevated bacterial load [120].

Whether other epigenetic marks, in addition to H3K4ac, contribute to coordinate *Drosophila* innate immune response is still a pending question. Moreover, the precise histone-modifying enzymes involved in the deposition of epigenetic marks in *Drosophila* remain to be identified. Similarly, whether other chromatin remodeling complexes, in addition to the SWI/SNF complexes, participate in a chromatin remodeling ballet to regulate the expression of immune genes in a dynamic manner remains to be explored.

Of note, mAkirin2 has recently been shown to be required for nuclear entry of proteasomes and turnover of some nuclear factors to control their short-lived activity [121]. Whether this function is independent of its chromatin regulation function remains to be explored.

## 6. Conclusions

The key to understanding the activation and resolution of the innate immune response seems linked to the characterization of the dynamics that are behind its modulation, particularly at the level of epigenetic regulation of gene expression [122]. Data point to a subtle system that controls the full activation of the pathway to occur only when it is required for a short period of time and allows partial activation in order to face small fluctuations in the environment. *Drosophila* remains an interesting model to explore this dynamic regulation and help address questions in the always important fields of NF-κB and cancer research.

## Figures and Tables

**Table 1:** **Modulators of the IMD pathway.** Table presenting the different proteins currently known to play a role in the activation or inhibition of the pathway. They are sorted by their localization in the cell.

Pathway Members	Sensors/Adaptors	Inhibitors
At the membrane level	PGRP-LC	PGRP-LB PGRP-SB PGRP-SC PGRP-LF Pirk
In the cytosol	PGRP-LE IMD FADD Dredd dIAP2 Eff Ben Uev1A TAB2 TAK1 IKKβ IKKγ Leswright Relish (Full)	Verloren dUSP36 Faf Trabid POSH PP4 CYLD Caspar Dnr1
In the nucleus	Relish (N-terminal) Hyd Akirin Bap60 Osa	dAP-1 Stat92E DSP1 HDAC1 Caudal Zfh1 H2Av
